# DEFINING A NOVEL HOMEOSTAT THAT SENSES THE BARRIERS OF PARENTAL REPRODUCTIVE-SPAN AND SETS PROGENY STRESS CAPACITY

**DOI:** 10.1093/geroni/igae098.3647

**Published:** 2024-12-31

**Authors:** Bennett Van Camp, Sean Curran

**Affiliations:** University of Southern California, Chandler, Arizona, United States; University of Southern California, Los Angeles, California, United States

## Abstract

The ability to respond to stressors quickly and adequately is a core component to survival, and dysregulation of these resistance pathways can be debilitating. The cytoprotective transcription factor SKN-1 plays a critical role in response to multiple types of stress that can impact Caenorhabditis elegans fitness (e.g., developmental timing, reproductive output). However, activation of SKN-1 is antagonistically pleiotropic, driving multiple age-dependent pathologies. For example, as compared to wildtype animals, SKN-1 activation in early adulthood provides enhanced resistance to oxidative stress, but greater sensitivity in midlife and an early loss of motility, imbalanced lipid homeostasis and diminished lifespan. Because the negative phenotypes associated with constitutive SKN-1 activation manifest at the end of reproduction, we use long-term parental age selection of late progeny (last quartile of total progeny) as a tool to investigate the differential health of early and late progeny across the reproductive span. Selection for >50 generations resulted in significant decreases of multiple SKN-1 age-related measures of health; most prominently, acute oxidative stress resistance, lipid homeostasis, movement, developmental timing, starvation resistance, and lifespan. Perhaps most surprising, acutely releasing the age selection resulted in instantaneous consequences on some, but not all, health parameters, of the immediate brood. This observation suggests the existence of a novel homeostatic set point that can bookmark the temporal boundaries of the parental reproductive span. These findings reshape the way we think about SKN-1 and the parental role in homeostatic control in offspring and provide additional details of its complex role in the regulation of healthspan.

